# Design and Construction of Chimeric VP8-S2 Antigen for Bovine Rotavirus and Bovine Coronavirus

**DOI:** 10.15171/apb.2016.014

**Published:** 2016-03-17

**Authors:** Khadijeh Nasiri, Mohammadreza Nassiri, Mojtaba Tahmoorespur, Alireza Haghparast, Saeed Zibaee

**Affiliations:** ^1^ Department of Animal Science, Faculty of Agriculture, Ferdowsi University of Mashhad, Iran.; ^2^ Institute of Biotechnology, Ferdowsi University of Mashhad, Iran.; ^3^ Department of Veterinary Medicine, Faculty of Veterinary Medicine, Ferdowsi University of Mashhad, Iran.; ^4^ Razi Vaccine and Serum Research Institute, Mashhad, Iran.

**Keywords:** Bovine Rotavirus, Bovine Coronavirus, Epitope, Expression, Recombinant protein

## Abstract

***Purpose:*** Bovine Rotavirus and Bovine Coronavirus are the most important causes of diarrhea in newborn calves and in some other species such as pigs and sheep. Rotavirus VP8 subunit is the major determinant of the viral infectivity and neutralization. Spike glycoprotein of coronavirus is responsible for induction of neutralizing antibody response.

***Methods:*** In the present study, several prediction programs were used to predict B and T-cells epitopes, secondary and tertiary structures, antigenicity ability and enzymatic degradation sites. Finally, a chimeric antigen was designed using computational techniques. The chimeric VP8-S2 antigen was constructed. It was cloned and sub-cloned into pGH and pET32a(+) expression vector. The recombinant pET32a(+)-VP8-S2 vector was transferred into E.oli BL21CodonPlus (DE3) as expression host. The recombinant VP8-S2 protein was purified by Ni-NTA chromatography column.

***Results:*** The results of colony PCR, enzyme digestion and sequencing showed that the VP8-S2 chimeric antigen has been successfully cloned and sub-cloned into pGH and pET32a(+).The results showed that E.coli was able to express VP8-S2 protein appropriately. This protein was expressed by induction of IPTG at concentration of 1mM and it was confirmed by Ni–NTA column, dot-blotting analysis and SDS-PAGE electrophoresis.

***Conclusion:*** The results of this study showed that E.coli can be used as an appropriate host to produce the recombinant VP8-S2 protein. This recombinant protein may be suitable to investigate to produce immunoglobulin, recombinant vaccine and diagnostic kit in future studies after it passes biological activity tests in vivo in animal model and or other suitable procedure.

## Introduction

Rotavirus group A is one of the main causes of diarrhea and gastrointestinal disease in humans, calves, sheep, swine.^1^ Bovine rotavirus group A (BRV) commonly occurs in calves 1-8 weeks of age.^2^ It is the main cause of mortality in calves and leads to increase treatment cost and reduces growth rate in calve.^3^ The BRV infects the mature enterocytes of the small intestine and causes pathological changes in the small intestine.^4^

The BRV group A belongs to genus Rotavirus in the family of Reoviridae and is composed of 11 segments of double stranded RNA surrounded by three concentric layers of protein.^5^ There are six viral proteins that are called VP1, VP2, VP3, VP4, VP6 and VP7. In addition, there are six nonstructural proteins (NSPs) that are only produced in cells infected by rotavirus, which are called NSP1, NSP2, NSP3, NSP4, NSP5 and NSP6.^6^ Based on serological cross-reactivities rotaviruses are classified into P serotype and G serotype and up to now 31 P and 23 G serotypes are identified that P[1], P[5], and P[11] of P and G6,G8 and G10 of G serotypes are the most frequent ones in calves.^7^

Proteins VP4 and VP7 from the outer layer are two independent antigens inducing neutralizing antibodies to the virus.^5^ It is believed that both VP4 and VP7 proteins are important for the development of rotavirus vaccine.^2^ The VP4 protein is a P type determinant whereas the VP7 protein is a G type determinant. The VP4 protein is a cytoplasmic protein, it is not glycosylated, and thus the protein produced in prokaryotes may mimic the native structure of the VP4 protein.^8^ The VP4 has no signal peptide at the amino terminus of the protein. The VP4 protein involved in some functions including: hemagglutination, cell penetration, neutralization and the determinant of virulence.^9^

The VP4 protein has a molecular mass of 88 kDa and it is presented as a series of spikes with 10-12 nm in length.^10^ The VP4 protein is cleaved by proteolytic enzyme, trypsin cleavage of V4 produces fragments VP8 (N-terminal region) and VP5 (C-terminal region),which have molecular mass of 28 kDa and 60 kDa, respectively. The VP8 protein has a hemagglutination domain.^11^ The cleavage site is located at position 241 and 247.^12^ Studies showed that VP8 and VP5 fragments can inhibit virus attachment to cells and the virus is neutralized by them.^13,14^ Some of studies have shown that some amino acids are conserved in the most rotavirus strains.^15^

Bovine coronavirus (BCV) is the major cause of diarrhea in neonatal calves and winter dysentery in cattle worldwide.^16^ It belongs to Coronaviridea family.^17^ The BCV contains a large single and positive-stranded RNA genome. It has structural proteins which are called nucleocapsid (N), tran-membrane (M), spike (S), small membrane (E), and hemagglutinin/esterase (HE).^18^

The S protein of BCV is a glycoprotein of 1363 amino acids and it is on the surface of the coronavirus virion that contains two regions, one of which is located at N-terminal (S1subunit) and the other located at C-terminal(S2 subunit). The S protein is cleaved in position 768 and 769 to two subunits by trypsin cleavage.^19^ It is involved in some functions such as induction of neutralizing antibody response,^20^ and binding to susceptible cell.^21^

Computational tools can be used for the prediction of B and T-cell epitopes for their use in antibody production, immunodiagnostics and epitope-based vaccine design.^22^ Bioinformatic approaches are relatively rapid and inexpensive. They can be used, at least in part to replace by experimental methods.^23^ Several epitopes prediction software programs are available. The first generation of these prediction tools performed base on motif-based algorithms,^24^ antigen primary amino acid sequence,^25^ 3D structure and other protein characteristics such as accessibility, hydrophilicity and flexibility.^26^

Because, the VP8 and S2 antigens induce protective immune responses against bovine rotavirus and coronavirus. On the other side the chimeric recombinant antigens are economical to introduce and produce an effective multivalent vaccine; the objective of the present study was the use of combined rotavirus-coronavirus antigens to design a multi-epitope antigen to investigate cloning and expression in prokaryote cell. Then, this antigen introduce to produce immunoglobulin, recombinant vaccine and diagnostic kit in future studies after it passes biological activity tests in vivo in animal model and or other suitable procedure.

In the present study, we used the bioinformatics tools to predict antigenic regions of the VP8 and S2 proteins in order to construct VP8-S2 chimeric antigen. The chimeric VP8-S2 antigen was cloned and sub-cloned into pGH and pET32a(+). Co-expression of the chimeric VP8-S2 antigen was investigated in prokaryote cell.

## Materials and Methods

### Strains, enzymes and Chemicals

The *E.coli* strain TOP10F' and BL21 CondonPlus (DE3) strains were provided from Novagen (USA). The plasmid pET32a(+) was purchased from Novagen. The restriction enzymes, Taq DNA polymerase and T4 ligase were purchased from Thermo. Bacterial culture media was purchased from Merck (Germany). The oligonucleotides were provided from Macrogen (South Korea). GeneJET Plasmid Miniprep Kit and GeneJET Gel Extraction Kit were from Thermo scientific (Fermentas). Expression vector pGH was obtained from GENEray (China)

### Bioinformatic tools for prediction antigenic regions

The nucleotide sequence of the VP8 (Fj598316) of G10P[11] genotype and S2 (NC_003045.1) gene were obtained from GenBank (http://www.ncbi.nih.gov/genbank/). For epitope prediction of the S2 protein, we chose conserved regions of S2 protein. B and T-cell epitopes of the proteins encoded by these genes were predicted using different servers and software like as: ABCpred, BepiPred, BCPred, SVMTrip and LEPS for B-cell prediction and IEDB, SYFPEITH, PropredI and Propred for T-cell prediction. Several supplementary criteria such as antigenicity, hydrophobicity, accessibility and flexibility were used in epitope characterization. The fragments that involve high density of immune-dominant epitopes were selected for each of the antigens.

Secondary structure was predicted using the improved self-optimized prediction method (SOPMA) software (http://npsa-pbil.ibcp.fr/cgi-bin/npsa_automat.pl?page=/NPSA/ npsa_sopma.html).^27^ Four conformational states (helices, sheets, turns and coils) of candidate genes were analyzed. Tertiary structure was predicted by various servers such as I-TASSER (http://zhanglab.ccmb.med.umich.edu/I-TASSER/) and Phyre (http://www.sbg.bio.ic.ac.uk/~phyre2/html/page.cgi?id=index).

The epitopes of the VP8 and S2 protein were screened for predicting their antigenicity using an online antigen prediction server VaxiJen v2.0 server (http://www.ddg-pharmfac.net/vaxijen/VaxiJen/VaxiJen.html). Furthermore, enzymatic degradation sites, Mass (Da) and pI were determined using the Protein Digest server (http://db.systemsbiology.net:8080/proteomicsToolkit/proteinDigest.html).

The fragments fused together by linker (G_4_S)_3_ to find the best epitope. This linker contains glycine and serine residues and it is a flexible linker. The gene sequence was codon optimized for expression in *E.coli* by GENEray. Codon optimization of sequence does not change amino acids coded by DNA, Hence could not affect adversely on selected epitopes.

### Enzymatic digestion pGH-VP8-S2 recombinant plasmid

The pGH-VP8-S2 vector containing the gene of insert flanked by *Bam*HI and *Xoh*I restriction site was transformed into *E.coli* strain TOP10F'. Then, the recombinant plasmids were cultured in LB medium that contained ampicillin antibiotic and was cultured for 16 h at 37°C in shaker incubator. GeneJET Plasmid Miniprep Kit (Fermentas) was used to purify recombinant plasmid. Plasmid DNA concentration was determined by NanoDrop ND1000 (Thermo Scientific, USA). The pGH-VP8-S2 recombinant plasmid was double digested by *Bam*HI and *Xoh*I enzymes. Double digest contained in total volume 25 µL: 13µL of recombinant plasmid, 10 units of each restriction enzyme, 2.5µL of 10x buffer R, and 0.5µL BSA and 8µL of dH_2_o. Digested products were checked by electrophoresis on 1% agarose gel.

### Construction pET32a(+)-VP8-S2 plasmid

The pET32a(+) vector was double digested by *Bam*HI and *Xoh*I enzymes. Then, the VP8-S2 fragment was ligated through T4 DNA ligase procedure, 16 h at 16°C into pET32a(+) vector, in order to construct the pET32a(+)-VP8-S2 expression plasmid. This construction was contained an internal 6x His tag and S-tag along with a N-terminal thioredoxin fusion protein. The recombinant pET32a(+)-VP8-S2 vector was transformed into *E.coli* strain TOP10F' cell and was grown overnight at 37°C on LB agar plates with ampicillin (100 μg/ml). Then, the recombinant plasmids were screened by PCR colony and digestion of Miniprep plasmid by *Bam*HI and *Xoh*I enzymes. The PCR colony carried out with pET T7 primers. For colony PCR, single colony was picked and resuspended in 100µL of ddH_2_O, then the tubes were boiled at 95°C for 10 min. Tubes were centrifuged for 5 min at 4000 rpm and 18 µL of the supernatant was used as template in PCR reaction and it was carried out in total volume of 25.2µL containing 1.5 mM MgCl_2_ (1.5 μL), 50 mM buffer 10X(2.5 μL), 0.2 mM of each of the 4 nucleotide dNTPs(2μL),100 pM of T7 primer(1 μL), 100 ng of DNA template(18 μL) and 0.2 U of *Taq* DNA polymerase. The PCR cycle conditions of an initial denaturation at 94°C for 8 min, followed by 34 cycle of 94°C for 30sec, 58°C for 45sec and 72°C for 45sec and final extension at 72°C for 10 min. GeneRuler^TM^ 1kb DNA Ladder(Fermentas) was used to compare the DNA fragment sizes. The amplified PCR fragment was separated by electrophoresis in a 1% agarose gel, stained with ethidiumboromide and visualized under a UV light and photographed with an UVidoc GEL Documentation System (UVitec, UK). Finally, the correctness of sequence was verified by DNA sequencing using T7 promoter (5'-TAATACGACTCACTATAGGG-3') and pET T7 terminator (5'- GCTAGTTATTGCTCAGCGG-3') primers.

### Gene expression of the VP8-S2 in E.coli

*E.coli* BL21 CondonPlus (DE3) harboring the recombinant pET32a(+)-VP8-S2 vector was grown in Luria broth (LB) culture supplemented with 100 μg/mL ampicillin and incubated overnight at 37°C in 150 rpm. Fresh LB liquid (50 ml) containing 100 μg/mL ampicillin was incubated by 5ml of pre-culture and it was incubated at 37°C in 150 rpm to reach OD_600:_0.6 (approximately 3 h). Then, culture was induced by 1mM IPTG and incubated at 37°C with shaking at 150 rpm for 6 h. The cells were harvested by centrifugation at 8000 rpm at 4°C for 10 min. The VP8-S2 expression was evaluated on 12% SDS-PAGE and visualized by Coomassie-blue staining.

### Protein solubility determination and protein purification 

The cell pellet was resuspended in lysis buffer (50mM NaH2PO4, 300mM NaCl, 10mM Imidazole, pH=8). Lysozyme was added at concentration of 1mg/ml for enzymatic digestion. It was incubated on ice for 30 min. The cells were disrupted by sonication for 10 min with 20s intervals between pulses. The cell was centrifuged in 12000 rpm at 4°C for 20 min. Supernatant and pellets were investigated to find where the supernatant contained the soluble VP8-S2 or insoluble VP8-S2 was present in the pellet. These samples were analyzed on 12% SDS-PAGE gel.

The VP8-S2 recombinant protein was purified based on manufacturer’s instructions Qiagen nickel- nitrilotriacetic acid (Ni-NTA) agarose Column (Qiagen, Hilden, Germany). In order to solubilize inclusion bodies, pellet was resuspended in buffer B (100 mM NaH_2_PO_4_, 10 mMTris-Cl, 8M Urea, pH=8) for 60 min on ice, lysate was centrifuged at 10000 rpm for 20 min at room temperature. We added 1ml of the 50% Ni-NTA slurry to 4 ml lysate and mixed it by shaking for 60 min. The lysate-Ni-NTA mixture was loaded into a column with the bottom outlet capped. The recombinant protein was washed once with 4ml of buffer C (100 mM NaH_2_PO_4_, 10 mMTris-Cl, 8M Urea, pH=6.3). The 6Xhis-tagged recombinant protein was eluted 4 times with 0.5 ml of buffer D (100 mM NaH_2_PO_4_, 10 mMTris-Cl, 8M Urea, pH=5.9) and followed by 4 times with 0.5 ml of buffer E (100 mM NaH_2_PO_4_, 10 mMTris-Cl, 8M Urea, pH=4.5). The recombinant VP8-S2 protein was further identified by SDS-PAGE analysis and concentration of protein was quantified by Bradford assay. This assay is based on the binding of the dye Coomassie blue G-250 to the protein and measure the absorbance at 595 nm (Bradford, 1976).

### Dot-blotting analysis of the recombinant VP8-S2 protein 

Dot blot analysis was carried out according to standard protocols.^28^ The recombinant VP8-S2 protein (2μg) was induced by 1mM IPTG, the extract of the transformed bacteria uninduced by IPTG and PBS were dot-blotted on nitrocellulose membrane. The membrane was immersed in 1% bovine serum albumin and was shaken into incubator for thirty minutes in the room temperature, it was washed with PBST (PBS, 0.1% (v/v) Tween) for two min, and then it was immersed in primary antibody (anti His-tag rabbit). It was diluted 1:500 for one hour on shaking incubator in the room temperature. After that, the nitrocellulose membrane was washed four times for five minutes each time in PBST, it was incubated with secondary antibody (anti-rabbit IgG conjugated to HRP) that was diluted 1:3000 for one hour on shaking incubator in the room temperature. The membrane was washed four times for five minutes each time in PBST. Color development was observed by adding diaminobenzidine dissolved in PBS and H2O2.

## Results and Discussion

### Prediction of epitopes for VP8 and S2 antigens

The B-cell and T-cell epitope predictions were successfully performed using different online software. For each of the software the highest score epitopes was selected as appropriate epitopes. Moreover, five epitopes were chosen as final epitopes by identifying epitopes which had most conserved sequences in all proposed epitopes (Table 1).

The chimeric VP8-S2 protein was identified as an antigen by the VaxiJen 2.0 server (threshold 0.5) with a final score of 0.59. The antigenicity of the final predicted epitopes shows in Table 1. The results of VaxiJen 2.0 analysis indicated that five predicted epitopes of the VP8-S2 gene were antigenic. The results of Protein Digest server analysis for determination of mass (Da), PI and enzymatic degradation site are shown in Table 2.

**Table 1:** Final epitopes after filtration and antigenicity ability of the predicted epitopes.

**Antigen**	**Number**	**Final epitope after filtration**	**VaxiJen score**
**VP8**	1	_8_QLLYNSYSVDLSDEITNIGAEK_29_	0.72*
**VP8**	2	_180_ADTQGDLRVGTYSNPVPNAVV_200_	0.57*
**VP8**	3	_223_GLPAMQTTTYVTPISYAIR_242_	0.92*
**S2**	4	_975_ATSASLFPPWSAAAGVPFYLNVQYR_999_	0.92*
**S2**	5	_1187_SGYFVNVNNTW_1198_	0.84*

* Probabl antigen

**Table 2:** Protein Digest analysis of the final epitopes.

**Epitopes**	**Mass(Da)**	**PI**	**Undigested enzyme**
_8_ **QLLYNSYSVDLSDEITNIGAEK**_29_	2472.69	3.92	Trypsin, Clostripain, Iodose , Proline_Endopept , Benzoate ,Cyanogen_Bromide, Trypsin K, Trypsin R
_180_ **ADTQGDLRVGTYSNPVPNAVV**_200_	2173.37	4.21	Cyanogen_Bromide, Iodose Benzoate, Staph Protease, Trypsin K
_223_ **GLPAMQTTTYVTPISYAIR**_242_	2083.43	8.59	Trypsin, Clostripain, Iodose Benzoate, Staph Protease, Trypsin K, Trypsin R, AspN
_975_ **ATSASLFPPWSAAAGVPFYLNVQYR**_999_	2714.08	8.63	Trypsin, Clostripain, Cyanogen_Bromide, Staph Protease, Trypsin K, Trypsin R, AspN,
_1187_ **SGYFVNVNNTW**_1198_	1300.39	5.24	Trypsin, Clostripain, Cyanogen_Bromide, Iodose Benzoate, Proline_ Endopept, Staph Protease, Trypsin K, Trypsin R, AspN

**Figure 1:**
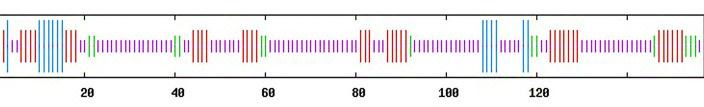
Secondary structure prediction results for the VP8-S2 protein. Blue: α helix; green: β turn; red: extended strand; and Purple: random coil.

**Figure 2:**
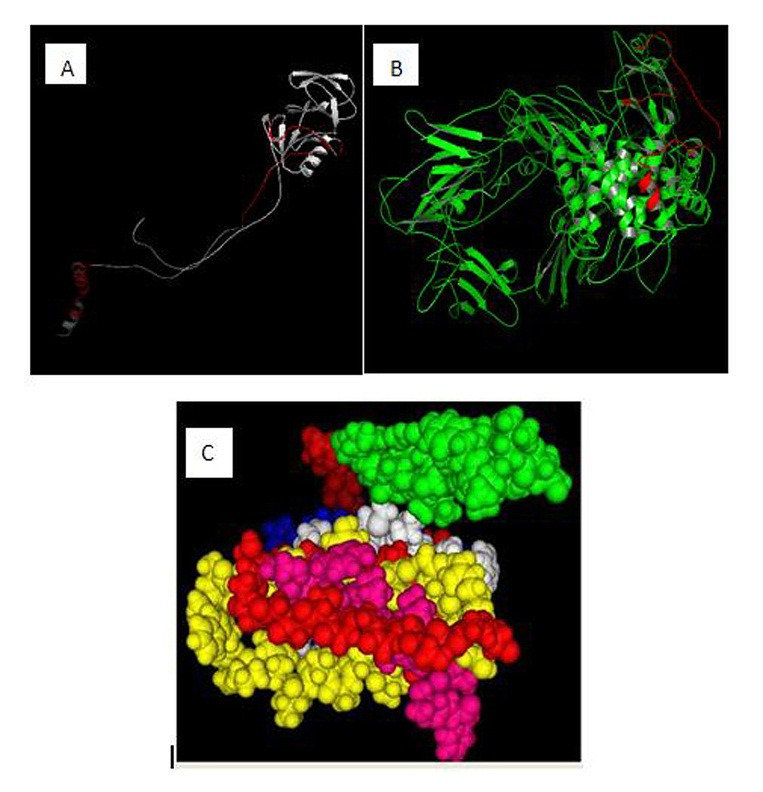
(A): Common predicted epitopes of the VP8 showed by red color, (B): Common predicted epitopes of the S2 proteins showed by red color, (C): the tertiary structure of the VP8-S2 protein.

To assess the antigenic features of the VP8-S2 protein, we predicted its secondary structure using SOPMA server. A greater proportion of extended strands and random coils present in the structure of the chimeric VP8-S2 protein (Figure 1). The results revealed that the proportion of random coils, β turns, α helices and extended strands (β folds) accounted for 58.60, 13, 14 and 38% of the secondary structure, respectively. The 3D structure of predicted epitopes with antigenicity ability were illustrated using 3D Ligand Site server (Figure 2). The 3D structure analysis also showed that all predicted epitopes located on the outside of the VP8 and S2 antigens molecule.

### Protein expression and purification analyses 

**Figure 3:**
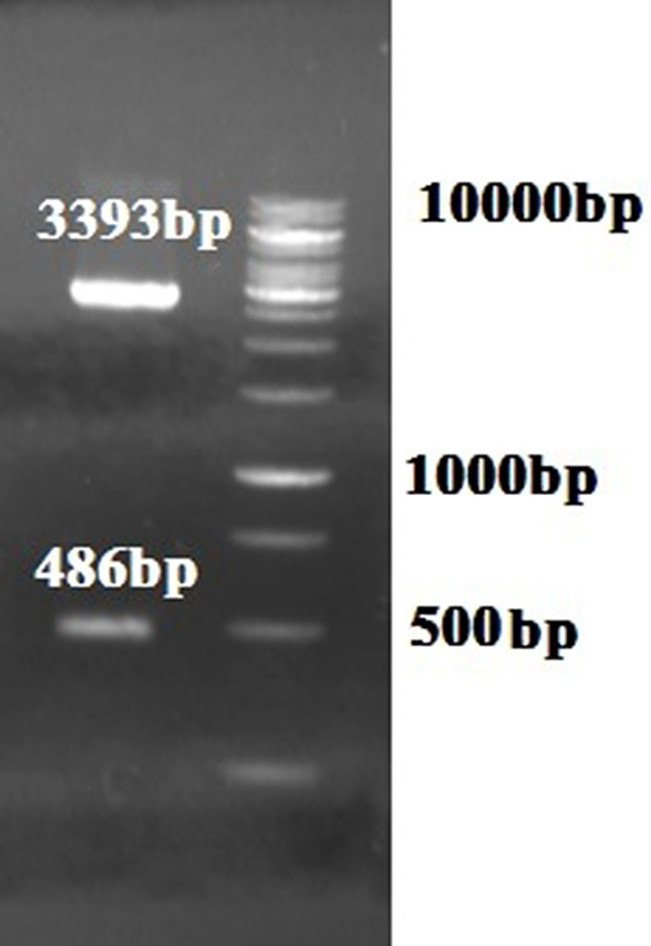
Digestion of the recombinant pGH-VP8-S2 plasmid (lane 1: 1kb DNA size marker and lane 2: digested recombinant plasmid).

The recombinant pGH-VP8-S2 plasmid was successfully transformed into TOP10F'. It was successfully digested with *Bam*HI and *Xoh*I enzymes. The presence of VP8-S2 fragment (486bp) and pGH vector (3393 bp) are shown in Figure 3.

**Figure 4:**
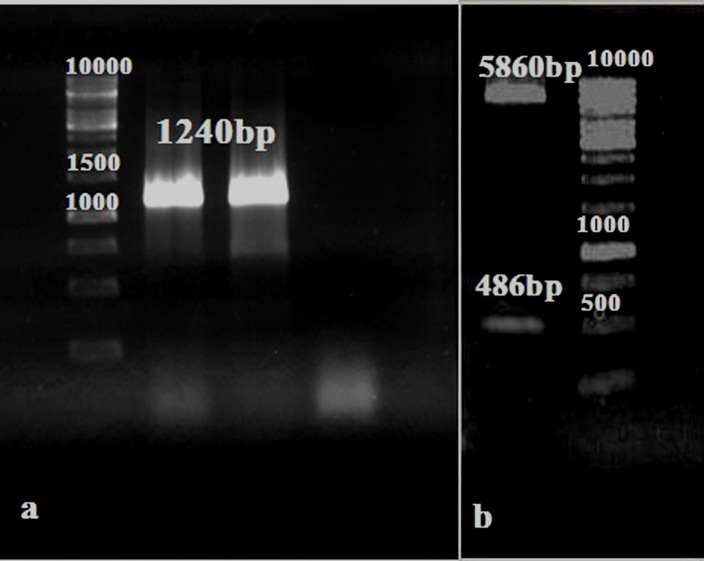
(a) Electrophoresis of the colony PCR of VP8-S2 gene (1240bp) by pET T7 primers (lane 1: negative control, lane 2 and 3: the VP8-S2 gene and lane 4: 1kb DNA size marker). (b) Digestion of the recombinant pET32a(+)-VP8-S2 vector (lane 1: 1kb DNA size marker and lane 2: digested pET32a(+)-VP8-S2 plasmid).

The chimeric VP8-S2 gene were successfully sub-cloned into expression plasmid pET32a(+). The results of recombinant colonies were verified using colony PCR by pET T7 primers (Figure 4a). The existence of VP8-S2 fragment in pET32a(+) were confirmed by enzymatic digestion (Figure 4b). The results of sequencing showed no changes in amino acid sequence of the VP8-S2 protein.

**Figure 5:**
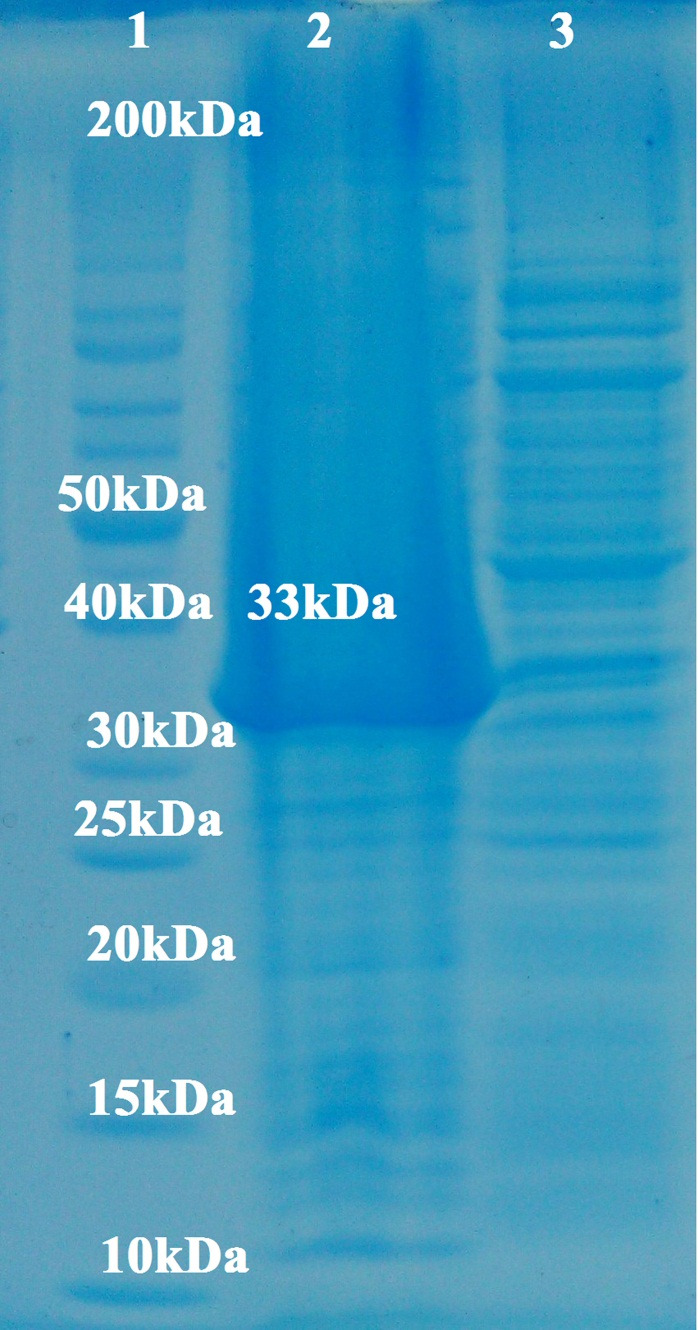
SDS-PAGE electrophoresis of the recombinant VP8-S2 protein (lane1: protein size marker 200kDa, lane 2: pellets induced by IPTG and lane 3: pellet uninduced by IPTG).

Expression of the recombinant VP8-S2 protein was checked by 12% SDS-PAGE electrophoresis by 1mM concentration of IPTG in two hours after induction (Figure 5). A protein 33 kDa (pET32(18.5kDa)+ VP8-S2(14.45 kDa)) could be detected in Coomassie blue staining. The results of expression analysis indicated that recombinant VP8-S2 protein could be expressed highly in *E.coli* cells. This vector is able to express a fusion protein with a 6-histidine tag at thrombin site and a T7 tag at the N-terminus. These additional amino acids increase to the size of the expressed protein near 18.5KDa.

**Figure 6:**
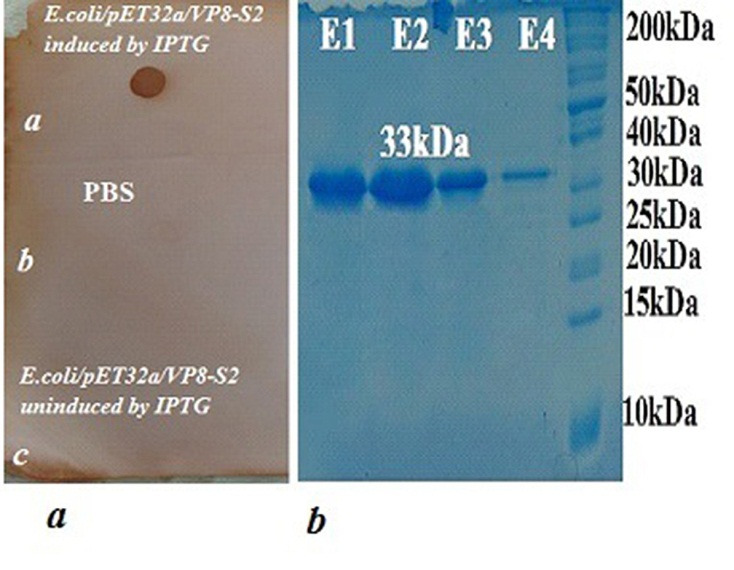
(a) Dot-blot analysis using anti-His tag antibody. (A). Brown dot shows the extract of transformed bacteria (*E.coli*/pET32a(+)/VP8-S2) induced by 1mM IPTG,(B)PBS and(C) the extract of the transformed bacteria uninduced by IPTG show as negative controls.(b) electrophoresis of SDS-PAGE analysis of the purification steps: (Lane 1, 2, 3 and 4): four fractions of the eluted proteins (E1, E2, E3 and E4). (Lane 5): protein molecular weight marker.

This recombinant protein was verified by dot-blot analysis. The result of dot-blot analysis using anti-His tag confirmed the existence of recombinant VP8-S2 protein (Figure 6a). The analysis of the supernatant and pellet of *Ecoli*BL21CodonPlus (DE3) cells by SDS-PAGE revealed that the expressed VP8-S2 protein was found in pellet. The recombinant VP8-S2 protein was successfully purified using Ni-NTA agarose Column (Figure 6b). The concentration of recombinant protein was calculated as 3 mg/ml.

The BRV and BCV are the most common viruses of diarrhea in neonatal calves, piglet and sheep. It leads to economic loss following treatment costs, decrease of growth rate and mortality would be detrimental.^29^ Studies showed that the most important pathogens in calf diarrhea are rotavirus, *Enterotoxigenic Escherichia coli*, coronavirus, salmonella and cryptosporidium.^30^ Maternal antibodies (colostrum) protect the neonatal offspring against rotavirus and coronavirus diarrhea for the first several days of life. Moreover, milk antibodies drop rapidly in cattle that often develop neonatal diarrhea after calves become infected with rotavirus and coronavirus.^31^ The development of vaccines or administration of milk supplements will be cased passive protection for the first several days of life against BRV and BCV.^32^

The development of epitope vaccines based on experimental research is very costly research which uses molecular biology and immunology technologies. The accuracy of this computational approach has been greatly enhanced using statistical methods.^33^ Many of researchers have used the VP8 and S2 antigens as a candidate vaccine in different expression schemes like baculovirus,^34^* E.coli*,^35,36^ and concluded that these antigens can be considered an immunogen in vaccine studies against the BRV and BCV.^37,7^

The main strategy in this study was to design a chimeric antigen (named VP8-S2) carrying various epitopes of the BRV and BCV for co-expression them into *E.coli.* Comprehensive bioinformatics analyses were conducted on candidate antigens by online B and T-cell epitopic prediction servers. We selected well-known online epitope prediction servers and a multi-method analysis approach to enhance the accuracy of epitopes prediction of the VP8-S2 antigen. The bioinformatics analysis of the VP8 and S2 antigens successfully predicted experimentally demonstrated epitopes. We used VaxiJen server for determining antigenicity of the chimeric VP8-S2 antigen because VaxiJen is the first server for alignment-independent prediction of protective antigens. It was developed to allow antigen classification based solely on the physicochemical properties of the protein irrespective of sequence length and the need for alignment. It identifies bacterial, viral and tumour antigens using different models.^38^ Linkers play an important role in displaying the epitopes on surface of fusion proteins.^39^ We used Glycine and Serine (GS_4_)_3_ residues from flexible linkers that could separate different domains in chimeric proteins and they have sufficient flexibility that may enhance protein expression level.^40^ Then chimeric VP8-S2 gene was constructed, it was cloned and sub-cloned into pGH and pET32a(+), respectively. The chimeric VP8-S2 gene was expressed in *E.coli* strain BL21 CondonPlus (DE3) and the recombinant VP8-S2 protein was produced appropriately. It purified using Ni-NTA agarose column for studying its immunogenic potentials in the future.

Mayameei et al^41^ (2010) revealed that the prevalence of rotavirus and coronavirus infection in diarrheic calves of dairy farms around Mashhad (Iran), were 26.98% and 3.17% respectively. Another study, major types of BRV in Tehran, Alborz and Qazvin of Iran were detected and bovine rotavirus was detected in 39.2% of total samples using ELISA kit. The results of this researchers showed that G10 of type G and P[11] of P type were the most prevalent. Also, the incidence of genotype combination G10P[11], G6P[5] and G6P[11] were 51.4%, 14.3% and 8.6%, respectively.^42^ In other studies, the tobacco mosaic virus vector containing the VP8 bovine rotavirus gene was used for expression of the recombinant VP8 protein and after intraperitoneal inoculation, antibody responses were detected against bovine rotavirus in mice, the mice protected against bovine rotavirus infection.^43^ Yoo et al^36^ (1997) expressed the VP8* protein of bovine rotavirus strain C486 in E. coli and examined potential of the recombinant VP8* protein for induction of neutralizing antibody responses in host animals. Their results suggest that the E. coli-produced recombinant VP8* protein can be an useful subunit vaccine candidate to prevent rotavirus infection in newborn calves. Favacho et^44^ (2006) synthesized VP8 cDNA of strain C486, amplified it by RT-PCR, it was successfully cloned and sub-cloned into pGEM-Easy plasmid and pET28(+) respectively, and it was expressed into *E.coli* BL21(DE3)pLysS. Also, they showed that the VP8ext providing a good source of antigen for the production of P type-specific immune reagents. In one study, Spike glycoprotein (S1 and S2 subunits) was expressed in Sodopterafugiperda insect cells. The result ofinfection of insect cells by the recombinant baculoviruses showed that the S1 and S2 subunits were expressed appropriately.^33^

The finding of this study was in agreement with the other findings that revealed *E.coli* expression system can be appropriate for high expression of the VP8 and S2 antigens of BRV and BCV, respectively.^36,44^ Also, the result of this current study was in agreement with the findings of investigators that reported the VP8 antigen has been expressed as fusion protein in insoluble form.^37,44-46^

## Conclusion

The results of this study indicated the recombinant VP8-S2 protein may be suitable to produce immunoglobulin, recombinant vaccine and diagnostic kit in future studies. This construction may be useful to be investigated as candidate for development of detection methods for simultaneous diagnosis of both infections and to reduce screening costs substantially after it passes biological activity tests in vivo in animal modeland or other suitable procedure. The next step of this research is whether the multi-epitope recombinant antigen can be stimulating to the humoral and cellular immune system.

## Acknowledgments

The authors thank Ferdowsi University of Mashhad for financial support of this study. The authors declare that there is no Conflict of Interests.

## Ethical Issues

Not applicable.

## Conflict of Interest

The authors report no conflicts of interest.
